# Editorial: Wearable Technology for Human Performance

**DOI:** 10.3389/fphys.2022.871159

**Published:** 2022-05-12

**Authors:** Colin K. Drummond, Beth Lewandowski, Carlo Massaroni

**Affiliations:** ^1^ Department of Biomedical Engineering, Case Western Reserve University, Cleveland, OH, United States; ^2^ National Aeronautics and Space Administration, Glenn Research Center, Cleveland, OH, United States; ^3^ Unit of Measurement and Biomedical Instrumentation, Departmental Faculty of Engineering, Università Campus Bio-Medico di Roma, Rome, Italy

**Keywords:** biomedical sensors, wearable sensors, human performance, physiological monitoring, physical monitoring, machine learning

Interest in measuring human performance has been increased in popularity and desire, driven by a realization that the growing need for quantitative validation of one’s health is now conveniently and inexpensively possible. Advances in semiconductor technology, materials development, and emerging algorithmic platforms (i.e., AI and machine learning) have dramatically increased quality and lowered the cost of biometric sensor technology. The impetus for this growing field is a new capability for tangible measurements of individual performance across multidisciplinary and diverse populations of people. The assessment of human performance and improvement can apply to anyone from the everyday person to an elite-level athlete and military personnel. The use of wearable sensor technology has provided an avenue for quantifying data that medical professionals and sports scientists have been seeking to analyze. The translational value of the wearables sensors field results from the combination of three factors:

• biomedical sensors becoming a commodity in the market,
• increasing sensitivity and selectivity of detected stimuli, and
• low-power and smaller sensor subsets.

While the market dictates the type of sensor to be utilized, the detected stimuli and sensor subsets necessitate the required fabrication strategy, which enables the detection of the biomarker or physiological stimuli of interest. The impact of translational value is thus governed by the ability to sync the three together to achieve the desired goal to have a direct effect on the patient.

This Research Topic features five papers that identify progress in product development as well as gaps in the technology of wearables for human performance monitoring. Collectively, these articles highlight beneficial applications of wearable technologies to improve safety and performance within occupational settings, diagnose and predict clinical events, and provide motivation towards improved self-health management. Measurements of stress level, sleep status, and physical exertion with wearable devices, combined with probabilistic models, provided predictions of human performance outcomes, including reaction time, executive function, and perceptuo-motor control. Changes in movement efficiency, derived via changes in acceleration data obtained from wearable accelerometers, provided quantifiable differences in the performance of manual labor tasks within hot and temperate environments. This quantifiable information can be used to identify the onset of fatigue or when there is an increased risk for injury. Understanding the physiological demands of activities that are expected to be performed during Mars missions by measuring heart rate, respiration rate, and heart rate variability with wearable devices allows for minimizing mission safety risks and aids mission planners in developing a plan extravehicular activity schedules.

Data obtained from smartwatches were used to develop a method for diagnosing bipolar depression and detecting the onset of a manic episode to provide care to patients in a more effective and timely manner. The use of a wearable biometric ring that provided feedback on daily activity level, the level of sleep quality obtained, recovery, and personalized education, encouragement, and feedback elicited a significant positive effect on the user’s healthy lifestyle. All of these examples of wearable technology applications provided benefits towards a goal of increased performance, safety, clinical treatment, or self-health; however, they also all had in common a challenge with individual variability. While acknowledging the existence of individual variability provides for the potential to develop customizable applications on an individual basis, it also presents a challenge when developing models and mathematical algorithms to fit a large segment of the population.

As evidenced in our Special Research Topic, wearable sensors are advancing from collecting consumer-quality wellness data to continuous clinical-quality physiological and physical data. Therefore, there are numerous challenges from a variety of perspectives ranging from the data quality required to ensure adequate measurement accuracy to the need to ensure the integrity of transmitted data and respect the user’s privacy. In the following years, we will see an increment in wearable customization, versatility, style, fabrics to make devices that people will be happy to attach to themselves. We will see new forms of wearables beyond the common smartwatch or ring we are already accustomed to seeing on many users’ wrists. The speedy technological developments producing a drastic reduction of costs and dimensions of devices, the possibility to measure new and unexplored variables, the on-sensor computational capabilities booming will push wearables to monitor human performances of users, workers, and athletes for long periods of time and under challenging conditions (during operation, in new environments even outside of terrestrial conditions). With new data otherwise unavailable under these conditions, the need to create common databases while respecting users’ privacy will become increasingly important. We expect to see increasing interest in this field. This abundance of real-world data will lead to new advanced algorithms based on artificial intelligence with tangible opportunities to identify new biomarkers related to human performance. This will make it possible to overcome the current limits on data-driven algorithms that should not contain or promote biases, which are inevitable when the sample of users is small and restricted to a particular geographic area, as well as when data are taken in the laboratory under certain conditions. Only in this way could we use data taken with wearable devices not only to provide vital signs, physiological quantities and predict measures but also to support evidence-based clinical and operational choices.

The articles presented in this Research Topic highlight the progress that has been made in enabling consumer recreational devices the ability to “crossover” into medical monitoring and diagnostics. First, the work of Hill and Caldwell provides insight into the challenges of field-based data Research Topic with wearables during demanding physical environments, providing insight on wearable use outside controlled laboratory settings. From a different perspective—but also examining the context of wearable use—the longitudinal study of Browne et al. points out the value of personalized education and feedback when seeking to influence behavioral change. Computational psychiatry is explored in the work of Llamocca et al. where smartwatch and self-reported data support machine learning algorithms assisting in improved accuracy of the diagnosis of bipolar depression. Research lead by Hostler et al. examines the significance of sensor placement in the assessment of injury risk. The results touch on the importance of fatigue assessment and suggest the roles wearable devices can play when translating wearable technology into practice. In the last paper, Brunyé et al. the challenges of modelling human performance prediction are tackled and in this work we again see how integrating human behavior parameters with wearable device data—while challenging from a modeling perspective—provides a meaningful role for machine learning to enhance human performance prediction. These five papers reflect encouraging trends in the convergence of wearable data, self-reported symptoms, and medical care, but the need remains for the continued leveraging of this work and the construction of the base of knowledge necessary for realizing maximum value from wearables. As reflected in these papers, there continues to be rich opportunities for collaborations between health professionals, data scientists, and engineers, and we hope this Research Topic is a step in that direction.

